# The safety and efficacy of biologic agents in treatment of systemic lupus erythematosus: A network meta-analysis

**DOI:** 10.12669/pjms.35.6.771

**Published:** 2019

**Authors:** Meng-Jun Tao, Ping Cheng, Lai-Run Jin, Jun Zhou, Wei Shi, Hui Peng, Liang Xu, Zhi Li, Hui Yuan

**Affiliations:** 1 Meng-Jun Tao, Department of Epidemiology and Biostatistics, School of Public Health, Wannan Medical College, Wuhu, PR China; 2 Ping Cheng, Administration Office of Education Cluster, Yijishan Hospital of Wannan Medical College,Wuhu, P.R. China; 3 Lai-Run Jin, Department of Epidemiology and Biostatistics, School of Public Health, Wannan Medical College, Wuhu, PR China; 4 Jun Zhou, Department of Epidemiology and Biostatistics, School of Public Health, Wannan Medical College, Wuhu, PR China; 5 Wei Shi, Department of Epidemiology and Biostatistics, School of Public Health, Wannan Medical College, Wuhu, PR China; 6 Hui Peng, Administration Office of Hospital Admission and Discharge, Yijishan Hospital of Wannan Medical College, Wuhu, P.R. China; 7 Liang Xu, Department of Rheumatology, Yijishan Hospital of Wannan Medical College, Wuhu, P.R. China; 8 Zhi Li, Department of Rheumatology, Yijishan Hospital of Wannan Medical College, Wuhu, P.R. China; 9 Hui Yuan, Department of Epidemiology and Biostatistics, School of Public Health, Wannan Medical College, Wuhu, PR China

**Keywords:** Atacicept, Belimumab, Biologic agents, Blisibimod, Epratuzumab, Network meta-analysis, Rituximab, SLE, Tabaluma

## Abstract

**Objective:**

Previous studies have shown that biologic agents out of the nine medicines might be beneficial for the treatment of SLE. The aim of this study was to evaluate the most effective medication of six biologic agents in treatment of SLE using network meta-analysis (NMA). The performance of these processes is ranked according to the results of this analysis.

**Methods:**

Multiple databases including PubMed, EMBASE and Cochrane Library was used to identify applicable articles and collect relevant data to analyzed by using STATA (13.0) software. The papers included in this study were divided into control group (placebo) and observation group (one of the six medicines).

**Results:**

A total of 21 eligible RCTs of biologic agents were identified, a total of 995 papers were included, and the results showed that the belimumab had the highest probability of being the most clinically efficacious intervention, with a surface under the cumulative ranking (SUCRA) curve of 75.0, was significantly superior (P < 0.05) to placebo alone. The blisibimod was the worst, with a SUCRA value of 29.4. The other biologic agents (atacicept, blisibimod, epratuzumab, rituximab, tabalumab) were insignificantly superior (P > 0.05) to placebo alone.

**Conclusions:**

Belimumab had the highest probability of being the best treatment for SLE compared with the other biologic agents (atacicept, blisibimod, epratuzumab, rituximab, tabalumab). The other biologic agents indicated an insignificant difference in efficacy for the treatment of SLE compared with placebo.

## INTRODUCTION

Systemic lupus erythematosus (SLE) is a chronic autoimmune disease, characterized by auto-antibody production, complement activation, and immune complex deposition.^1^ Auto-antibodies mediate inflammation and various organs damaged through immune complex formation.^2^ We already knew that many factors such as infection environment, immunity and many other factor are closely related to cause this disease that adaptive immune system has been the focus of many studies.^3^ Current treatment strategies rely heavily on corticosteroids. This in turn leads to a cascade of events including increase in infections and malignancies, limit in immunosuppressives, long standing over reliance on corticosteroid therapy.^4^ Currently available treatment has involved the use of anti-inflammatory or immunosuppressive non-steroidal anti-inflammatory drugs to deal with different situation.^5^ This conventional treatment can be associated with organ damage and not completely effective in many patients, which highlighting a huge need in the area of SLE therapeutics.^6^

In recent years, an increased understanding of the etiopathogenesis has led to development of biologic agents for SLE has been pressing, which may significantly improve the task of treating SLE.^5^ Among the biologic agents (atacicept, belimumab, blisibimod, epratuzumab, rituximab, tabalumab) for SLE, it is important to evaluate the efficacy of six biologic agents by systematic review and meta-analysis. Although the efficacy of the multiple biologic agents which used to treat SLE was acceptable, there was no direct comparison between the two interventions. Network meta-analysis (NMA) was an upgrade from traditional meta-analysis (TMA). This study may improve a useful guide for selection of medication treatments for SLE.

## METHODS

### Search strategy:

The databases searched for this study included PubMed, EMBASE and Cochrane Library, before 4^th^ September 2018, using atacicept, belimumab, blisibimod, epratuzumab, rituximab or tabalumab and SLE. Through literature traceability, we read relevant reviews to view their references and other ways to trap, as much as possible to find all relevant information.

### Inclusion and exclusion criteria:

The inclusion criteria were as following:

a. Randomized controlled trials
b. Both the experimental group and the control group were SLE patients
c. Data acquisition in around 52 weeks
d. The data of efficacy or adverse reactions are complete and can be analyzed by NMA
e. Document language was English.

The exclusion criteria were as following:

a. Animal experiments, cross-experimental studies
b. Case reports, systematic reviews
c. Comparison before and after drug treatment, or no data available for analysis
d. The patients with other disease included LN.

### Efficacy evaluation criteria:

Outcome indicators included SRI-4, SRI-6, because of different situations.

The SRI-4 was defined as the following:

a. ≥ 4-point reduction in SELENA-SLEDAI score compared with baseline
b. No worsening (<0.3-point increase from baseline) in Physician’s Global Assessment (PGA)
c. No new British Isles Lupus Assessment Group (BILAG) A organ domain score or two new BILAG B organ domain scores vs baseline).

The SRI-6 was defined as the following:

a. ≥6-point reduction in SELENA-SLEDAI score compared with baseline
b. No worsening (<0.3-point increase from baseline) in Physician’s Global Assessment (PGA)
c. No new British Isles Lupus Assessment Group (BILAG) A organ domain score or two new BILAG B organ domain scores vs baseline).

All analyses were adapted from previous published work. Thus, no ethical approval and patient consent were required.

### Data extraction and quality evaluation:

Literature search and extraction were performed independently by two reviewers, based on the inclusion and exclusion criteria, include the following:

a. Characteristics of the publication
b. Data quality of the publication
c. Result indicator selection.

### Statistical analysis:

By using commands of the network package in statistical (13.0), the network, evidence contribution, predictive interval (PrI), funnel and ranking plots were constructed. The efficacy of the intervention was ranked based on the surface values under the cumulative ranking (SUCRA) curve. The selected indicator was the count data, and *OR* is used as the combined effect, with a confidence interval (*CI*) set to 95%. A value of *P* < 0.05 was considered to be statistically significant.

## RESULTS

**Fig. 1:**
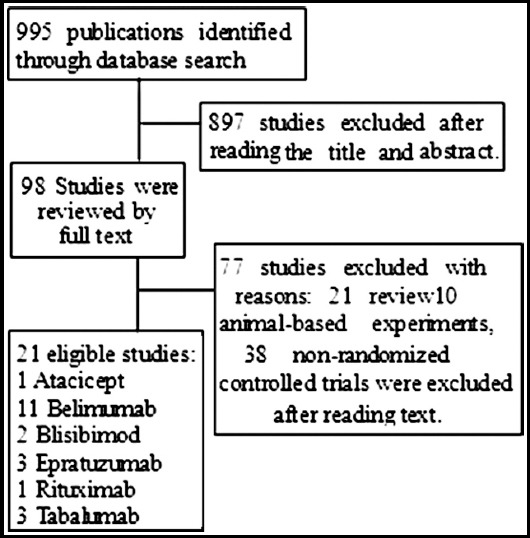
The selection details of included publications.

A total of 21 RTCs involving 12276 patients were ultimately included in this study. Fig. 1 show the select detail of publication includes. The basic characteristics of publications are presented in Table-I.

**Table I:** Basic information of included studies in the network meta-analysis.

Medicine	Author	Country of patients	A	B	C	D	E	F	G	During (weeks)	outcome	Jadad quality score
Belimumab	Ronald F van Vollenhoven	Sweden, USA, Spain, UK	287	589						52	1	3
Belimumab	A Doria	USA, Europe, Asia	141	435						52	1	4
Belimumab	Fengchun Zhang	China, Japan, Soutd Korea	217	446						52	1	4
Belimumab	RICHARD A. FURIE	UK, USA	86	235						52	1	3
Belimumab	A. Doria	USA, Italy, Japan, Brazil, Netderlands, Nortd Carolina	108	248						52	1	4
Belimumab	Yoshiya Tanaka	Japan	20	39						52	1	4
Belimumab	Richard Furie	Asia, USA, Europe	275	544						52	1	4
Belimumab	Susan Manzi	USA, Canada, Italy, Mexico	287	589						52	1	3
Belimumab	Ellen M	USA	86	235						52	1	4
Belimumab	William Stohl MD	USA, Europe, Australia and Asia	280	556						52	1	4
Belimumab	Vibeke Strand	USA, UK, Spain, Brazil,	287	578						52	1	2
Atacicept	Joan T. Merrill	Latin America, Asia, Soutd Africa, Europe, UK, USA	100		206					52	1	3
Epratuzumab	Megan E. B. Clowse	Nortd America, Latin America, Europe, tde Middle East, India, Korea, China	512			1017				52	1	4
Epratuzumab	Daniel J Wallace	USA, UK, China	38			189				52	1	4
Epratuzumab	Daniel J. Wallace	USA,UK	40			65				52	1	4
Tabalumab	D A Isenberg	USA, Asian	381				757			52	1	4
Tabalumab	J T Merrill	USA, Canada, Mexico, Central America, Soutd America, Asia-Pacific, Africa/Middle East Europe	376				752			52	1	4
Tabalumab	Yoshiya Tanaka	Japanese	15				15			52	1	4
Rituximab	JT Merrill	UK,USA	88					169		52	1	3
Blisibimod	Joan T Merrill	Belarus, Brazil, Colombia, Georgia, Guatemala, China, Korea, Singapore, Malaysia, Mexico, Russia, Sri Lanka, Thailand and the Philippines	197						245	52	2	4
Blisibimod	R A Furie	Argentina , Brazil, Chile, Colombia, China, India, Mexico, Peru, the Philippines, USA	269						277	52	2	4

1: SIR4; 2: SIR6. Placebo: A; Belimumab: B; Atacicept: C; Epratuzumab: D; Tabalumab: E; Rituximab: F; Blisibimod: G.

### Network meta-analysis

**Fig. 2:**
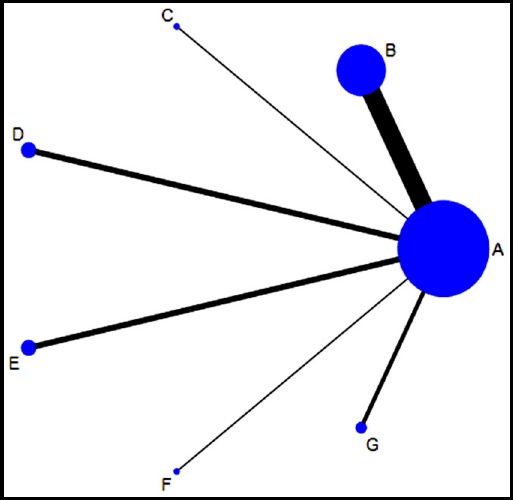
Network plot of different targeted therapies for the treatment of SLE ***Abbreviations:*** Placebo: A, Belimumab: B, Atacicept: C, Epratuzumab: D, Tabalumab: E, Rituximab: F, Blisibimod: G

*Network plot of six different medicines:* Of the 21 publications studies on the biologic agents for SLE with belimumab were the most frequent, while those on atacicept and rituximab were least. The highest number of subjects was belimumab, while atacicept has the lowest number of this studies (Fig. 2). The size of the points in the network graph is proportional to the number of subjects, while the thickness of the line is proportional to the number of studies.

### Evidence contribution plot

**Fig. 3:**
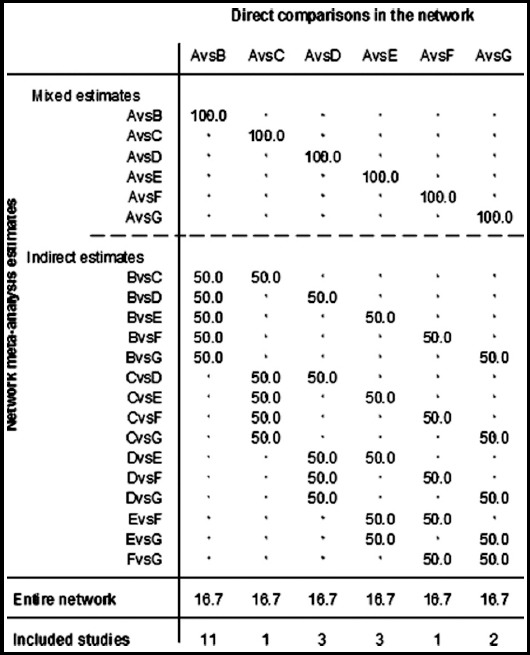
The effect of comparing the results of different control measures. ***Abbreviations:*** Placebo: A, Belimumab: B, Atacicept: C, Epratuzumab: D, Tabalumab: E, Rituximab: F, Blisibimod: G

The direct comparison of placebo alone and belimumab had a 100% effect on the combined results. The direct comparison between placebo and belimumab had a 50% effect on the indirect comparison between belimumab and atacicept. The direct comparison of placebo and belimumab had a 16.7% effect on the results of the NMA (Fig. 3).

### Predictive interval plot

**Fig. 4:**
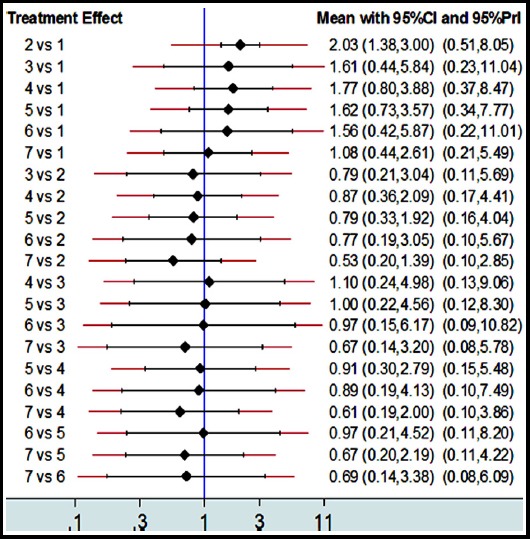
Network estimates of mean *OR*, their 95% *CIs* and prediction intervals (red extensions) ***Abbreviations:*** Placebo: 1, Belimumab: 2, Atacicept: 3, Epratuzumab: 4, Tabalumab: 5, Rituximab: 6, Blisibimod: 7

In this study, it is showed that the pooled *OR* and 95% *CI* of SLE improvement compared with placebo were 2.03 (1.38-3.00) for belimumab, 1.61 (0.44-5.84) for atacicept, 1.77 (0.80-3.88) for epratuzumab, 1.62 (0.73-3.57) for tabalumab, 1.56 (0.42-5.87) for rituximab, 1.08 (0.44-2.61) for blisibimod, respectively, which indicates an insignificant difference in efficacy except for belimumab. The comparison between other medicines is showed in Fig. 4.

### Publication bias

**Fig. 5:**
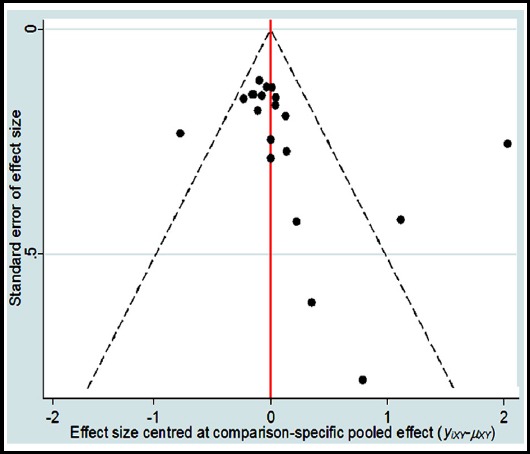
Funnel plot for publication bias of different medicines.

Regarding publication bias, all results in the study are basically symmetrical (Fig. 5). The probability distribution for each treatment is ranked for their efficacy in SLE according to SUCRA values (Table-II and Fig. 4). The order of SUCRA values for different biologic agents was as follows: belimumab (75.0); epratuzumab (62.0); tabalumab (57.1); atacicept (55.1); rituximab (52.6); blisibimod (29.4) placebo (18.7); From this study, the belimumab had the highest probability of being the best treatment in biologic agents.

**Table II:** SUCRA of SLE treatments.

Treatment	SUCRA (%)	Pr Best	Mean Rank
Belimumab	75	20.9	2.5
Epratuzumab	62	17.2	3.3
Tabalumab	57.1	13.1	3.6
Atacicept	55.1	24.1	3.7
Rituximab	52.6	21.7	3.8
Blisibimod	29.4	3	5.2
Placebo	18.7	0	5.9

**Fig. 6:**
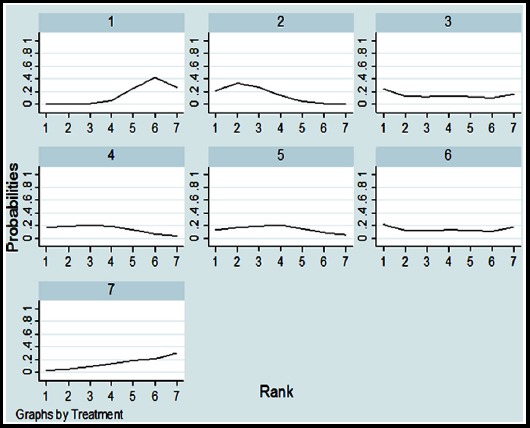
SUCRA for the cumulative probabilities. ***Abbreviations:*** Placebo: 1, Belimumab: 2, Atacicept: 3, Epratuzumab: 4, Tabalumab: 5, Rituximab: 6, Blisibimod: 7

## DISCUSSIONS

The study analyzed six biologic agents for SLE in 21 randomized controlled trials. These results showed that the belimumab had the highest probability of being the best treatment compared with other biologic agents (atacicept, blisibimod, epratuzumab, rituximab, tabalumab), according to network meta-analysis by network diagram makes it more intuitive. Belimumab was more effective highest SUCRA value and highest probability of being the best treatment option, while other medicines indicated an insignificant difference in efficacy.

The SLE is caused by immune complexes depositing on organs and extensive injury were caused.^7^ The patients with SLE are characterized by BCR-initiated signaling and IL-6 production, including alter in B cell subset distribution.^8^ Therefore, the main clinical strategy for treating SLE was blocking the immune cells stimulating cytokine that affects the development of SLE. The main goal of current treatment strategies, which are not ideal in terms of efficacy and safety, was to use a limited dose of corticosteroids to prevent injury and maintain stable disease control.^9^-^11^ Biologic agents are being developed to enhance therapeutic efficacy, reduce disease exacerbation and toxicities. Currently, drugs for the SLE treatment evolved from all the patients recommended antimalarial to nonsteroidal anti-inflammatory drugs, glucocorticoids and combination of biologic agents.

It was divideded into nine kinds according to its mechanism of action on the following:

a. B cell therapies;
b. Proteasome inhibitors;
c. Inhibition of B/T cell costimulation;
d. Targeting Pdc;
e. Targeting cytokines and their receptors;
f. Targeting the interferons;
g. Targeting the kinases of the intracellular machinery;
h. Targeting the sphingosine-1-phosphate;
i. Other mechanisms of action.^12^

This study analyzed six kinds of biologic agents (atacicept, belimumab, blisibimod, epratuzumab, rituximab, tabalumab), belonging to B cell therapies, to exclude classical immunosuppressive agents, and belimumab may be the most effective.

Belimumab is recombinant human immunoglobulin (Ig) G1-λmAb, which molecular weight of ~147 kDa.^13^ It specifically binds to soluble B-lymphocyte stimulator (BLyS), prevents its interaction with other receptors, inhibits B cell apoptosis, stimulates B cells to differentiate into immunoglobulins.^14^ BlyS and its receptors (TACI, BCMA and BAFF-R) remain the focal point of therapeutic targets for SLE therapy as autoimmune B cell stimulation and maturation play a major role in the disease onset.^15^ In mouse models of systemic lupus erythematosus, BLyS inhibition delays lupus onset, while in clinical trials, belimumab reduces the number of peripheral CD20 + B cells, which is predominantly naive, significantly reduces SLE disease activity, flare rates and prednisone dose in seropositive patients.^16^-^18^

In this study, we focused on SIR response, while adverse reactions also occurred in clinical trials, including headache, fever, nausea, diarrhea and other side effect. The causes of deaths include serious infections, heart disease and suicide. Hypersensitivity reactions may occur, such as immediate withdrawal, and appropriate treatment.^19^

In addition, the biologic agents except for belimumab (atacicept, blisibimod, epratuzumab, rituximab, tabalumab) were insignificantly superior to placebo. These approaches biologic agents of B cell therapies include: block BLyS, modulate B cell signaling, neutralise soluble BLyS, induce depletion of B cells, block with all three forms of BLyS.^20^-^24^ The causes of the results are unclear, while biologic agents in treatment of systemic lupus erythematosus are still a long way to go whatever in safe or efficacy.

### Limitations of study:

In this study, the lack of uniform standards for efficacy evaluation and inconsistent quality of the original publication used may have some effect on the strength of the proposed argument. The results may be affected by inconsistently literature quality, great heterogeneity inherent to SLE, different ethnicity and sample size. Future studies involving high quality RCT and large sample size are needed.

## CONCLUSIONS

Biologic agents except for belimumab (atacicept, blisibimod, epratuzumab, rituximab, tabalumab) indicated an insignificant difference in efficacy for the treatment of SLE compared with placebo. Belimumab had the highest probability of being the best treatment for SLE compared with the other biologic agents (atacicept, blisibimod, epratuzumab, rituximab, tabalumab).

### Author`s Contribution:

**MJT** designed the study and conceived the survey.

**LRJ and LX** collected epidemiological data.

**ZL and HP** sorted the data.

**JZ and LX** were involved the fieldwork.

**MJT and PC** conducted the analysis and wrote the first draft of the manuscript. The first two authors contributed equally to this work and are considered co-first authors.

**Hui Yuan** evaluated the results and revised the manuscript, takes responsibility for the integrity of the research.
